# Sickness absence after work accidents and post-traumatic stress among white-collar workers in the retail and wholesale industry; a longitudinal Swedish cohort study

**DOI:** 10.1186/s12889-024-19865-0

**Published:** 2024-09-03

**Authors:** Kristin Farrants, Kristina Alexanderson

**Affiliations:** Division of Insurance Medicine, Department of Clinical Neuroscience, Karolinska Institutet, Stockholm, SE-171 77 Sweden

**Keywords:** Sick leave, Disability pension, Work accidents, Post-traumatic stress disorder, White-collar workers, Retail and wholesale industry, Population-based

## Abstract

**Background:**

Most studies about accidents and about PTSD, respectively, have been conducted either on blue-collar workers, or on the entire working population. There are very few such studies on white-collar workers.

**Aim:**

To examine diagnosis-specific sickness absence (SA) and disability pension (DP) after a work accident or PTSD, respectively, among white-collar workers in the private retail and wholesale industry.

**Methods:**

A prospective population-based cohort study of all 192,077 such workers aged 18–67 (44% women) in Sweden in 2012, using linked microdata from nationwide registers. We identified individuals who had secondary healthcare due to work-related accidents (*n* = 1114; 31% women) or to PTSD (*n* = 216; 79% women) in 2012–2016. Their average number of net days of diagnosis-specific SA (in SA spells > 14 days) and DP were calculated for 365 days before and 365 days after the healthcare visit.

**Results:**

35% of the women and 24% of the men had at least one new SA spell during the 365 days after healthcare due to work accidents. Among women, the average number of SA/DP days increased from 14 in the year before the visit to 31 days the year after; among men from 9 to 21 days. SA days due to fractures and other injuries increased most, while SA days due to mental diagnoses increased somewhat. 73% of women and 64% of men who had healthcare due to PTSD had at least one new SA spell in the next year. Women increased from 121 to 157 SA/DP days and men from 112 to 174. SA due to stress-related disorders and other mental diagnoses increased the most, while DP due to stress-related diagnoses and SA due to musculoskeletal diagnoses increased slightly.

**Conclusions:**

About a quarter of those who had secondary healthcare due to work accidents, and the majority of those with such healthcare due PTSD, had new SA in the following year. SA due to injury and mental diagnoses, respectively, increased most, however, SA/DP due to other diagnoses also increased slightly. More knowledge is needed on factors associated with having or not having SA/DP in different diagnoses after work accidents and among people with PTSD.

**Supplementary Information:**

The online version contains supplementary material available at 10.1186/s12889-024-19865-0.

## Introduction

Sickness absence (SA) and disability pension (DP) have consequences for the worker, their family, the employer, the insurer, and society as a whole.

Although there is a conception that the work environment in retail and wholesale is relatively safe compared to industries such as mining, agriculture, and construction, due to the large size of the sector, work accidents and SA in this industry still affects a substantial part of the labour market [1, 2]. Statistics from the Swedish Work Environment Authority show that, in the retail and wholesale sector, there were nearly 5 occupational accidents per 1000 workers that led to days on SA among women and approximately 7 per 1000 workers among men in 2022, which is neither among the highest nor the lowest among all industries in Sweden [3]. In the EU, 12.5% of non-fatal occupational accidents in 2020 and 2021 took place within the retail and wholesale sector [4].

Sickness absence (SA) due to work accidents are of considerable concern: approximately one third of SA days due to injuries are due to work accidents [5] and about one quarter of reported work accidents lead to SA spells exceeding 14 days, according to the Swedish Work Environment Authority [3]. Falls are the most common work accident leading to SA/DP among women, among men it is having lost control over tools or vehicles [3].

A study from the USA found that there are more days away from work due to occupational accidents in the retail and wholesale industry than in many of the recognized high-risk sectors mentioned above [1], and further, that they have a higher rate of serious work accidents and accidents than the private sector as a whole [1, 6]. Accidents at work are a reasonably common as well as preventable cause of SA/DP [7], and, among white-collar workers, injury diagnoses are the third most common cause of SA/DP days, after mental and musculoskeletal diagnoses [8, 9]. Work accidents can have consequences for physical and mental health, and can also, in some cases, lead to post-traumatic stress disorder (PTSD) [10, 11]. Work-related accidents have in common with other traumatic experiences that they are unpredictable and constitute a psychological threat [10]. The diagnostic criteria of PTSD include that the individual has been part of or witnessed a traumatic event, and because of that has clinically significant suffering or worsened functioning at least one month after the event, and suffer symptoms from the following four domains: intrusive symptoms, avoidance, negative cognitive changes or negative affect, and hyperactivation [12]. Post-traumatic stress due to traumatic experiences at work is more common in the retail and wholesale industries than in many other industries [1, 13]. There is some debate about whether PTSD should be considered an injury or a mental disorder [14]. PTSD is currently mainly treated in secondary healthcare in Sweden, although mild PTSD can also be treated in primary healthcare [12]. A diagnosis of PTSD diagnosis can be given if the symptoms affect the patient’s function or quality of life [12]. In most cases, the primary healthcare physician refers patients to secondary healthcare, although self-referral is also possible.

Most studies regarding SA/DP in conjunction with PTSD are done on healthcare professionals, police, or firefighters [13, 15]. However, a study from the USA showed that most of those who had sought healthcare due to PTSD after work-related incidents were cashiers or salespeople [13]. The authors also found that about half of the cases involved armed robberies, while 38% involved other situations of threats or violence [13]. So far there are only a few studies regarding SA/DP in conjunction with PTSD, however, they indicate that it is very common with SA/DP after traumatic experiences at work [13, 16, 17]. A small study based on self-reported data on threats and violence within the retail and wholesale industry found that the risk of PTSD was more than twice as high among those who had experienced a robbery at work, however, there were no differences regarding depression among those who had and hadn’t experienced such robberies [18]. Most who had experienced a robbery at work reported that they had not been sickness absent afterwards [18].

Most studies about work accidents and about PTSD have been done either on blue-collar workers, or on the entire working population. There are very few such studies on white-collar workers. While most who are in customer-facing jobs are blue-collar workers, there are also white-collar workers who deal with customers and who face a risk of a work accident. Therefore, it is also of interest to gain knowledge about SA/DP following work accidents and PTSD among white-collar workers. Nevertheless, to the best of our knowledge, there are no studies about SA and DP following work accidents or PTSD among white-collar workers in the retail and wholesale industry. Occupational accidents are unexpected and often traumatic [10]. It is important to gain knowledge on the occurrence of SA/DP following work-related accidents for the sake of prevention efforts. Moreover, irrespective of the reason (work-related or not) for a diagnosis, the possible effects on the employee’s work capacity need to be handled at work, e.g., by the employer and the individual. For this reason, it is important to gain knowledge on SA and DP in relation to different diagnoses. In this study, we focused on work-related accidents and on PTSD, respectively.

**The aim** was to examine diagnosis-specific sickness absence (SA) and disability pension (DP) after a work accident and post-traumatic stress disorder (PTSD), respectively, among white-collar workers in the retail and wholesale industry.

## Methods

This is a population-based longitudinal cohort study of SA and DP after secondary healthcare due to work accidents or due to PTSD among privately employed white-collar workers in the retail and wholesale industry in Sweden.

### Data and study population

We used data from four nation-wide Swedish administrative registers, linked at individual level by use of the Personal Identity Number (PIN, a unique ten-digit number assigned to all residents in Sweden) [19, 20]:

1) The Longitudinal Integration Database for Health Insurance and Labour Market Studies (LISA by Swedish acronym) held by Statistics Sweden: to identify the source population and for information on age, sex, country of birth, type of living area, family situation, educational level, income, occupational code according to the Swedish Standard for Occupational Classification (SSYK by Swedish acronym, the Swedish version of the International Classification of Occupations, ISCO), and branch of industry according to the Swedish Standard for Industrial Classification (SNI by Swedish acronym) in 2012–2016;

2) MicroData for Analysis of the Social Insurance database (MiDAS) held by the Swedish Social Insurance Agency: for information on SA spells > 14 days and DP (dates, extent (25%, 50%, 75%, or 100% of ordinary work hours), and main diagnosis for 2010–2016);

3) the National Patient Register held by the National Board of Health and Welfare: for information on visits to specialised in- or outpatient healthcare (that is, to secondary healthcare) (dates, diagnosis according to the International Classification of Diseases, Tenth Revision (ICD-10) [21]); and.

4) the Cause of Death Register held by the National Board of Health and Welfare: for information on date of death.

The source population was all those who were aged 18–67 years and registered as living in Sweden all of 2012, had an occupational code according to SSYK that indicated a white-collar occupation, were employed at a private sector company in the retail and wholesale industry according to SNI, and during 2012 had income from work, parental benefits, and/or SA/DP that amounted to at least 7920 SEK (that is, 75% of the necessary income level to qualify for SA benefits from the Social Insurance Agency). The limit of 75% of the minimum income to qualify for SA benefits was set since in many cases SA benefits is about 75% of the work income; without this adjustment, people with low incomes and long-term SA might have fallen below the minimum income level to be included in the study. Those who were employed in the public sector, self-employed, or who had full-time DP all of 2012 were excluded, while those who had SA or partial DP were included. This gave a cohort of 192,077 individuals.

### Public sickness absence insurance in Sweden

All people living in Sweden aged ≥ 16 years with an income from work or unemployment benefits, who due to disease or injury have a reduced work capacity, are covered by the national public SA insurance providing SA benefits. After a first qualifying day, the employer pays sick pay for the next 13 days of a SA spell, thereafter, SA benefits are paid by the Social Insurance Agency. Self-employed have more qualifying days. Unemployed individuals receive SA benefits from the Social Insurance Agency after the first qualifying day. A physician certificate is required after 7 days of self-certification. In this study, data on SA with benefits from the Social Insurance Agency were used. SA spells < 15 days were not included in the study, so as not to introduce bias regarding those who might have been unemployed part of the year. All residents in Sweden aged 19–64 years, whose work capacity is permanently or long-term reduced due to disease or injury, can be granted DP from the Social Insurance Agency.

Both SA and DP can be granted for part- or full-time (25, 50, 75, or 100% of ordinary work hours). SA benefits cover 80% and DP benefits 64% of lost income, both up to a certain level. We used net days of SA/DP so that partial days of SA/DP were combined, e.g., two days of part-time absence for 50% were summed to one net day. The first 14 days of the included SA spells were counted as being of the same extent as day 15 for the purpose of calculating net days. Mean number of SA/DP net days/calendar year were calculated for each individual.

### Secondary healthcare for work accidents and post-traumatic stress disorder

Secondary healthcare for work accidents was defined as having a hospitalisation or a physician outpatient secondary healthcare visit with an external cause code that indicated that the individual was engaged in work when the accident occurred at any point during 2012–2016. Since external cause codes are mostly used in combination with injury diagnoses, 90% of these visits had some kind of injury as the main diagnosis (ICD-10 S00-S99 or T00-T88).

Secondary healthcare for PTSD was defined as having a visit in secondary outpatient or a hospitalisation with post-traumatic stress (F43.1) as either main or secondary diagnosis. Since visits and stays due to PTSD were not recorded with external cause codes, there was no way of knowing whether the PTSD diagnosis was related to the patient’s work.

If an individual had more than one visit or stay in secondary healthcare due to work accident in the period 2012–2016, we chose the date of the first such visit or hospitalisation, i.e., the index visit. The same was done for PTSD.

### Diagnosis-specific sickness absence/disability pension

Diagnosis-specific SA/DP was categorised into the following diagnosis groups: mental diagnoses (ICD-10 codes F00-F99 and Z73), musculoskeletal diagnoses (M00-M99), injuries (S00-T98 and V01-Y98), cancer (C00-D48), cardiovascular diagnoses (I00-I99), pregnancy-related diagnoses (among women: O00-O99 and N96), or other diagnoses (all other diagnoses, including missing, excluding Z00-Z99). In the analyses related to work accidents, we separated out SA due to fractures (S02, S12, S22, S32, S42, S52, S62, S72, S82, S92, T02, T10) from other injuries (all other S00-T79, V01-Y98). In the analyses regarding PTSD, we separated out SA due to stress-related diagnoses (F43) from other mental diagnoses (F00-F42, F44-F99, Z73).

We calculated the proportion of individuals with incident SA or DP during the 365 days after the first visit or stay in out- or inpatient secondary healthcare, as well as the mean number of net days of SA and DP in the above-mentioned diagnoses during 365 days prior to and the 365 days after the date of the first visit or stay in secondary healthcare for work accident or PTSD, respectively. We also calculated crude and mutually adjusted odds ratios (OR) and 95% confidence intervals (95% CI) of associations between the sociodemographic variables described above and having SA/DP during the year following the work accident among those who had a work accident, using logistic regression. For these analyses, some categories were combined if the number of individuals with SA/DP in any category was < 10, in order to preserve anonymity and statistical power. These analyses were not conducted for those with PTSD due to low numbers of individuals.

## Results

Table 1 shows the sociodemographic characteristics of the cohort in 2012. There was a slightly higher proportion of men (55.56%) and more than half were aged 35–54.

The majority among both women and men lived in large cities (Stockholm, Gothenburg, or Malmö), and were born in Sweden. A small proportion (5.60% of women and 9.21% of men) had only elementary education, while 44.89% of women and 37.10% of men had at least some college/university education. The majority were married/cohabiting and having children < 18 years living at home. More than twice the proportion of women (9.40%) than men (3.72%) were single parents with children living at home.

**Table 1:** Sociodemographic and work-related information on the study cohort of privately employed white-collar workers in the retail and wholesale industry, at baseline in 2012

	Total			Women		Men	
	*n*		%	*n*	%	*n*	%
All	192,077		100	85,356	100	106,721	100
*Sex*							
Women	85,356		44.44				
Men	106,721		55.56				
*Age*							
18–24 years	8145		4.24	4485	5.25	3660	3.43
25–34 years	40,881		21.28	20,277	23.76	20,604	19.31
35–44 years	60,739		31.62	26,864	31.47	33,875	31.74
45–54 years	50,160		26.11	20,759	24.32	29,401	27.55
55–64 years	29,607		15.41	11,986	14.04	17,621	16.51
65–67 years	2545		1.32	985	1.15	1560	1.46
*Type of living area*							
Large city	99,445		51.77	46,242	54.18	53,203	49.85
Medium-sized town	60,893		31.70	25,431	29.79	35,462	33.23
Small town or rural	31,739		16.52	13,683	16.03	18,056	16.92
*Educational level (years)*							
Elementary (0–9 years)*	14,612		7.61	4778	5.60	9834	9.21
High school (10–12 years)	99,554		51.83	42,259	49.51	57,295	53.69
University/college (> 12 years)	77,911		40.56	38,319	44.89	39,592	37.10
*Country of birth*							
Sweden	175,508		91.37	76,709	89.87	98,799	92.58
Other Nordic country	4243		2.21	2324	2.72	1919	1.80
Other EU-25	3457		1.80	1685	1.97	1772	1.66
Rest of the world**	8869		4.62	4638	5.43	4231	3.96
*Family situation*							
Married/cohabiting without children at home		25,595	13.33	11,344	13.29	14,251	13.35
Married/cohabiting with children at home		96,068	50.02	40,530	47.48	55,538	52.04
Single without children		58,416	30.41	25,457	29.82	32,959	30.88
Single with children at home		11,998	6.25	8025	9.4	3973	3.72

*Including missing (*n* = 349). **Including missing (*n* = < *10).*

### Work accidents

Among all, 1114 people (0.55%) had specialist out- or inpatient healthcare due to work accidents; 343 women (0.40%) and 771 men (0.72%). Table 2 shows proportions with SA/DP in the year after the accident, ORs and 95% CIs of having SA/DP in the year after the accident. Of those who had secondary healthcare for work accidents, 35% of the women and 24% of the men had at least one new SA spell during the 365 days after the outpatient visit or hospital stay. A slightly higher proportion of women (35%) than men (24%) had SA/DP after their work accident. Those aged 55–67 had the highest proportion of individuals with SA/DP (38%), and those aged 25–34 had the lowest (23%), closely followed by those aged 35–44 years (23%). A slightly smaller proportion of those who were married/cohabiting with children at home had SA/DP following their work accident (26%) than individuals in other family situations. Otherwise, the distribution of SA/DP was quite similar in all groups; either just under or just over 30%.

Most associations from the logistic regression over having SA/DP in the year following the work accident were not significant in neither the crude nor adjusted model, with two exceptions: men had a lower likelihood of having SA/DP (adjusted OR 0.57; 95% CI 0.42–0.76), and those aged 55–67 were more likely to have SA/DP (adjusted OR 2.13; 95% CI 1.34–3.38).

**Table 2:** Numbers and proportions (%) of those with secondary healthcare due to a work accident who had new SA/DP in the following year, as well as crude and mutually adjusted odds ratios (OR) and 95% confidence intervals (CI) of SA/DP in the year after the healthcare visit

	*n* with SA/DP		% with SA/DP	Crude OR (95% CI)	Adjusted OR (95% CI)
**All**	305		27.38		
*Sex*					
Women	119		34.69	Ref.	Ref.
Men	186		24.12	0.60 (0.45–0.79)	0.57 (0.42–0.76)
*Age*					
18–24 years	22		30.99	1.48 (0.84–2.61)	1.12 (0.60–2.09)
25–34 years	58		22.66	0.97 (0.65–1.43)	0.90 (0.60–1.36)
35–44 years	73		23.25	Ref.	Ref.
45–54 years	91		29.26	1.37 (0.96–1.95)	1.39 (0.96–1.99)
55–67 years	61		37.65	1.99 (1.32–3.01)	2.13 (1.34–3.38)
*Type of living area*					
Large city	119		29.46	Ref.	Ref.
Medium-sized town	110		25.94	0.84 (0.62–1.14)	0.86 (0.63–1.18)
Small town or rural	76		26.57	0.87 (0.62–1.22)	0.88 (0.62–1.26)
*Educational level (years)*					
Elementary (0–9 years)*	30		26.32	1.13 (0.83–1.53)	1.22 (0.89–1.69)
High school (10–12 years)	196		28.24	1.03 (0.63–1.67)	1.04 (0.62–1.73)
University/college (> 12 years)	79		25.82	Ref.	Ref.
*Country of birth*					
Sweden	275		27.64	Ref.	Ref.
Not Sweden	30		25.21	0.88 (0.57–1.37)	0.86 (0.55–1.36)
*Family situation*					
Married/cohabiting without children at home		42	31.34	1.35 (0.89–2.05)	0.99 (0.61–1.59)
Married/cohabiting with children at home		128	25.25	Ref.	Ref.
Single without children		113	28.39	1.17 (0.87–1.58)	1.21 (0.87–1.69)
Single with children at home		22	29.33	1.23 (0.72–2.10)	1.03 (0.60–1.79)

**Fig. 1:**
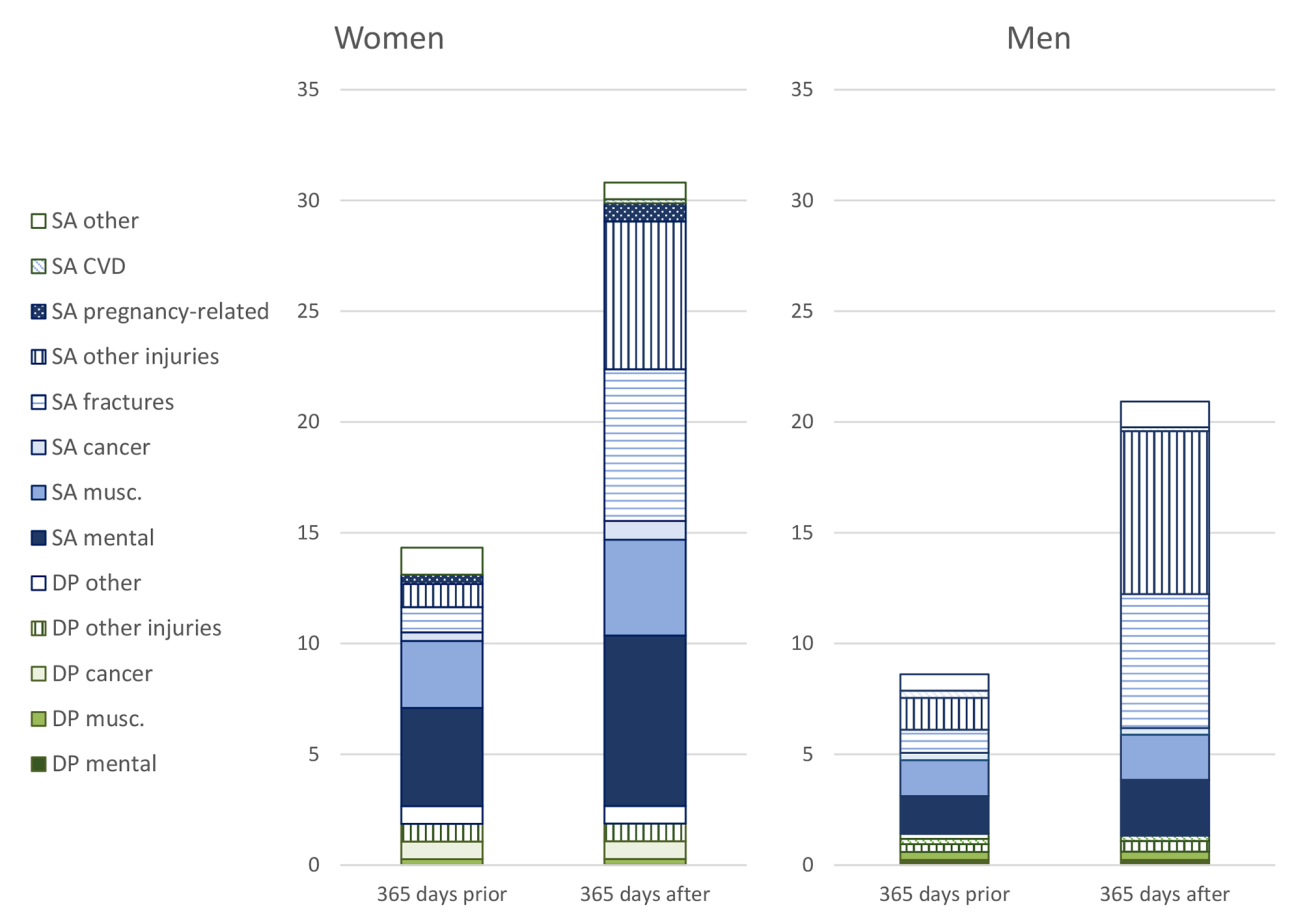
Mean number of net days of diagnosis-specific sickness absence (SA) and disability pension (DP) per person during the 365 days before and 365 days after a first visit to secondary out- or inpatient healthcare for a work injury, for women and men

Figure 1 and Supplementary Table 1 show the mean number of SA and DP net days per person in different diagnosis groups, 365 days prior to and 365 days following the first visit to secondary out- or inpatient healthcare due to a work accident (including the date of the first visit) among women and men. The year prior to their first such healthcare visit, women had an average of 14 days of SA/DP in total, and men an average of 9 days. The year following the first healthcare visit, women had an average of 31 days and men 21 days. About 14 of these days were due to fractures and other injury diagnoses among both women and men. The number of SA days increased by more than the number of DP days.

Mental and musculoskeletal diagnoses accounted for a majority of the SA and DP days among women and a plurality among men in the year prior to the healthcare for the work accident. However, even before the first visit to specialised healthcare due to the work accident, fractures and other injury diagnoses accounted for a sizeable minority of days. The year after the first healthcare visit, fractures accounted for the plurality of days among women, followed by other injury diagnoses, whereas for men, the order was the reverse. The mean number of SA days was higher in the year after the index healthcare visit due to work accident than in the year before. The number of SA days due to fractures and due to other injuries increased the most, but SA due to mental diagnoses also increased somewhat. The number of DP days did not increase substantially.

There were 171 women (0.20%) and 45 men (0.04%) who had secondary out- or inpatient healthcare due to PTSD, for a total of 216 individuals (0.11%). A slightly higher proportion of those who were born outside Europe had secondary healthcare for PTSD (0.56%) compared to those born in Sweden (0.08%), other Nordic countries (0.03%), or other EU-25 (0.20%).

Of those who had secondary healthcare for PTSD, 73% of women and 64% of men had at least one new spell of SA or DP during the 365 days following the date of the first visit or stay.

**Fig. 2:**
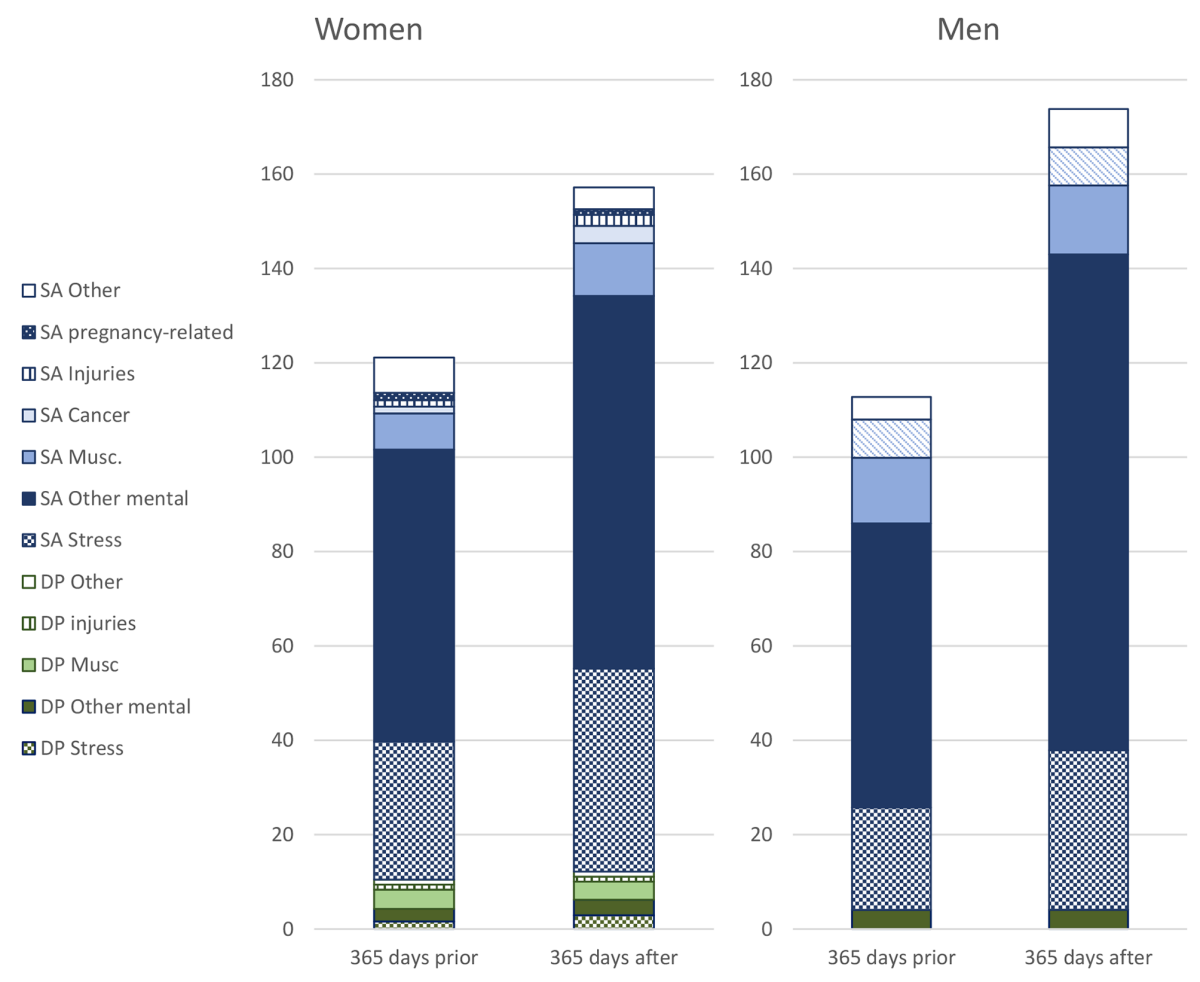
Mean number of net days of diagnosis-specific sickness absence (SA) and disability pension (DP) per person during the 365 days before and 365 days after a first visit to secondary out- or inpatient healthcare for post-traumatic stress disorder for women and men

Figure 2 and Supplementary Table 2 show the mean number of SA and DP net days per person in the different specific diagnosis groups, 365 days prior to and 365 days following this date (including the date of the first healthcare visit) among women and men. These individuals had a substantially higher average number of days than those with healthcare due to work accidents: an average of 121 days for women and 112 days for men. The following year women had an average of 157 days and men 174 days.

Other mental diagnoses accounted for a majority of SA-days in the year before the visit for both women and men, followed by SA due to stress-related diagnoses. In the year after the first visit, SA due to stress-related and other mental diagnoses increased the most, but even DP due to stress-related diagnoses increased slightly, as did SA days due to musculoskeletal diagnoses.

## Discussion

In this cohort study of SA and DP after specialized healthcare due to PTSD or due to work accidents, respectively, we found that the majority of those who had healthcare due to PTSD and a sizeable minority of those with healthcare due to work accidents had SA/DP within the following year. Women in the PTSD group had on average 120 SA/DP days and the men 115 SA/DP days in the 365 days before the healthcare visit date, and 160 days for women and 175 days in the year after that date. Women with such healthcare due to work accidents had on average 15 DA/DP days before the healthcare visit date and the men 10 days, and 30 days after for women and 20 days for men. Most SA/DP days were in diagnosis categories corresponding to the healthcare diagnosis (stress-related SA for those with PTSD, fractures and other injuries for those with work accidents), but a sizeable number of days were also with other mental and musculoskeletal diagnoses. Women and individuals aged 55–67 years were more likely to have SA/DP in the year after healthcare due to work accidents, however, no other significant differences were found.

Compared to all individuals in the retail and wholesale industry, both individuals with work accidents and PTSD had more SA and DP days in the year before the first respective secondary healthcare visit [22] – and this was particularly the case for individuals with PTSD. Even before the first index healthcare visit, individuals with work accidents had a higher number of net days with injury diagnoses, and those with PTSD with stress-related diagnoses, than in the general population of white-collar workers in the retail and wholesale industry [22]. This could be due to individuals seeking primary healthcare first, and having their SA diagnosis set by primary healthcare, before visiting secondary healthcare. However, it could also be that the morbidity that led to their prior SA increased their vulnerability to work accidents or PTSD. Pre-existing conditions have previously been found to be associated with the number of SA days after a work accident [23].

Both those who had PTSD and those who had work accidents had a higher number of SA and DP days after the visit to secondary healthcare than before: while this increase was larger for those with a work accident, those with PTSD already had much higher numbers. Unlike regarding work accidents, we have no information on whether the PTSD was work-related or not, however, our analyses show that many people with PTSD have work-related consequences, in terms of work incapacity, also before they received secondary healthcare for their PTSD. Work accidents and PTSD share some similarities, as they are both the result of disruptive events [10, 11], and that for workers, the consequences of these disruptive events need to be managed within the workplace. Our results indicate that both those who have work accidents and PTSD might need more support to manage the consequences of their respective disruptive event in order to prevent SA/DP or facilitate a quick return to work.

For those with work accidents, the number of SA days with mental and musculoskeletal diagnoses also increased after the index healthcare visit. Musculoskeletal injuries such as back pain are common types of injuries that can result from overexertion, and SA spells due to musculoskeletal diagnoses are often of long duration and high cost [1]. That mental diagnoses increased after the healthcare visit could be due to reactions to the accident, but it could also be pre-existing mental disorders that were identified by the physician.

Even though the proportions were very small, the proportion of men with work accidents was twice as high as that among women. Post-traumatic stress, conversely, was more common among women, even though it was even rarer than a work accident. Previous research has also found that men have a higher rate of work accidents [24]. This is likely due to differences in exposure to hazards in different occupations [24, 25], biological sex differences [25], and in recognizing musculoskeletal disorders as work accidents in women. There are also differences in the exposures between women and men in the same occupation, due to women and men not performing the same tasks, even in the same occupation [25], largely due to the strong numerical gender-segregation of jobs [26, 27]. We also found that women had more SA/DP days, both before and after the index healthcare visit for work accident, while for PTSD, women had more SA/DP days before the visit for PTSD, but fewer days after. Previous research has found that, in general, while women have a higher risk of SA, the number of SA days per person with SA is similar for women and men [22, 28]. Similarly, a previous study found that women are more likely to have PTSD, also when accounting for differences in exposure to potentially traumatizing events [29]. On the other hand, one study has suggested that women respond to treatment faster [30], although another study found the opposite [31]. It is also worth noting that there is often a difference between recovering from symptoms, and recovering one’s work capacity, and there is very little known about gender differences in work capacity recovery and return to work among those on SA [32, 33]. More studies are needed about gender differences in work capacity recovery in general, and in relation to PTSD specifically.

We found very few differences in the likelihood of SA/DP in the year following secondary healthcare due to a work accident, by any of the sociodemographic factors included. This could be due to the low number of individuals who had such healthcare (1114 individuals, of which 305 individuals had SA/DP). However, in many cases, the estimates were also relatively close to 1, indicating that other factors than sociodemographic factors are more important in determining whether an individual who has had a work accident requires SA/DP, possibly related to type and severity of the injury and type of work tasks. Previous research has found that, generally, more men have work accidents than women [34], however, women tend to have more days lost from work when they have a work accident [35]. This is in line with both our results regarding the likelihood of being on SA/DP after a work accident, as well as the mean number of days with SA/DP. Regarding age, previous research is less clear-cut, but a systematic review concluded that while younger workers were more likely to have a work injury than older workers, older workers were more likely to have fatal or severe accidents [34]. A study from Spain found that older workers on SA after an occupational accident tended to have longer duration of SA than younger workers [36]. Our study complements these studies by also showing that they were more likely to have SA/DP after a work accident.

All individuals in this study had white-collar occupations. However, a few other studies have shown occupational differences in the incidence of work accidents and related SA, also between different types of white-collar occupations [7, 37], as well as differences between retail and wholesale [1, 38] and types of retail [1, 6, 38]. These differences might be related to the work tasks required and the possibilities to perform the tasks with reduced work capacity [37]. One study also found poor concordance between work status and disability status [39]. All of this means that it is of interest to study incidence and duration of SA after work accidents and after PTSD, respectively, by occupation and sub-sector in more detail.

This is a first study of SA and DP following work accident and PTSD, respectively, among privately employed white-collar workers. More detailed studies are needed regarding potential associations and sub-groups differences. Comparisons with white-collar workers in other industries or blue-collar workers in retail and wholesale are also warranted.

## Strengths and limitations

The strengths of this study include the use of high-quality nationwide register data, with linked microdata on all white-collar employees in the retail and wholesale industry, without drop-outs or loss to follow-up, that information on SA/DP was register-based, i.e., not based on self-reports subject to recall bias, and that we could include information on SA/DP both before and after the index visit to secondary healthcare.

Limitations were that we only had information from secondary healthcare. Especially regarding secondary healthcare due to PTSD, the number of individuals who had any such healthcare was small (216 of approx. 190 000; 0.11%). It is plausible that more individuals had PTSD but were treated in primary healthcare or other forms of healthcare, or not at all; and the same might also be true for work accidents. However, it can also be seen as a strength that we only included those with more severe conditions. The small number of individuals with work accidents might have impacted the analysis of SA/DP after healthcare due to work accidents, resulting in wide confidence intervals and a few significant results.

While the use of register data in many ways comprise a strength of the study, one limitation of this approach is that we were not able to include factors that are not routinely collected in the registers, such as BMI, lifestyle factors (such as tobacco or alcohol use), or factors related to work environment or occupational health and safety at the workplaces.

We didn’t include self-employed individuals. There have been many contradictory results regarding whether self-employed individuals have better, worse, or similar health status as employees [40], and this has to the best of our knowledge not been considered for work accidents nor PTSD.

## Conclusion

About haa quarter of those who had secondary healthcare due to work accidents, and the majority of those who had thus with PTSD, had new SA spells following such healthcare. SA due to injury and mental diagnoses, respectively, increased most, however, SA/DP due to other diagnoses also increased slightly. More knowledge is needed on factors associated with having or not having SA/DP in different diagnoses after work accidents and PTSD.

### Electronic supplementary material

Below is the link to the electronic supplementary material.

Supplementary Material 1

## Data Availability

The used data cannot be made publicly available due to privacy regulations. According to the General Data Protection Regulation, the Swedish law SFS 2018:218, the Swedish Data Protection Act, the Swedish Ethical Review Act, and the Public Access to Information and Secrecy Act, these types of sensitive data can only be made available for specific purposes that meets the criteria for access to this type of sensitive and confidential data as determined by a legal review. Professor Kristina Alexanderson (Kristina.alexanderson@ki.se) can be contacted regarding the data.
