# Supplementary material for: Sickness absence after work accidents and post-traumatic stress among white-collar workers in the retail and wholesale industry; a longitudinal Swedish cohort study
Source: BMC Public Health. 2024 Sep 3;24:2389. doi: 10.1186/s12889-024-19865-0 (PMC11370135; doi:10.1186/s12889-024-19865-0)
Supplement: Supplementary file 1 — Supplementary Material 1 [file 12889_2024_19865_MOESM1_ESM.pdf]

Supplementary Table 1: Mean number of SA and DP net days in the year before and the year after specialist healthcare due to a work accident, among privately employed white-collar workers in retail and wholesale, for women and men.

	Women		Men	
Diagnosis	365 days prior	365 days after	365 days prior	365 days after
DP mental	0.00	0.01	0.24	0.24
DP musc.	0.27	0.27	0.36	0.37
DP cancer	0.80	0.80	0.00	0.00
DP fractures	0.00	0.00	0.00	0.00
DP other injuries	0.80	0.80	0.36	0.48
DP CVD	0.00	0.00	0.24	0.24
DP other	0.80	0.80	0.23	0.00
SA mental	4.44	7.69	1.70	2.52
SA musc.	3.02	4.33	1.62	2.05
SA cancer	0.39	0.85	0.34	0.30
SA fractures	1.14	6.84	1.04	6.04
SA other injuries	1.05	6.67	1.44	7.35
SA pregnancy-related	0.42	0.82	-	-
SA CVD	0.00	0.18	0.33	0.18
SA other	1.22	0.75	0.74	1.16

Supplementary Table 2: Mean number of SA and DP net days in the year before and year after a first specialist healthcare visit due to PTSD, among privately employed white-collar workers in retail and wholesale, for women and men.

	Women		Men	
	365 days prior	365 days after	365 days prior	365 days after
DP Stress	1.60	2.92	0.00	0.00
DP Other mental	2.67	3.29	4.06	4.10
DP Musc	4.07	3.85	0.00	0.00
DP injuries	1.07	1.07	0.00	0.00
DP Other	1.07	1.07	0.00	0.00
SA Stress	29.27	43.04	21.71	33.82
SA Other mental	61.86	78.97	60.20	105.08
SA Musc.	7.68	11.17	13.92	14.58
SA Cancer	1.46	3.67	0.00	0.00

SA Injuries	1.35	2.35	0.00	0.00
SA CVD	0.00	0.00	8.11	8.11
SA pregnancy-related	1.56	1.20	-	-
SA Other	7.48	4.61	4.77	8.11
